# Bifactor Models for Predicting Criteria by General and Specific Factors: Problems of Nonidentifiability and Alternative Solutions

**DOI:** 10.3390/jintelligence6030042

**Published:** 2018-09-07

**Authors:** Michael Eid, Stefan Krumm, Tobias Koch, Julian Schulze

**Affiliations:** 1 Department of Education and Psychology, Freie Universität Berlin, Habelschwerdter Allee 45, 14195 Berlin, Germany; stefan.krumm@fu-berlin.de (S.K.); julian.schulze@fu-berlin.de (J.S.); 2 Methodology Center, Leuphana Universität Lüneburg, 21335 Lüneburg, Germany; tobias.koch@leuphana.de

**Keywords:** bifactor model, identification, bifactor(*S*-1) model, general factor, specific factors

## Abstract

The bifactor model is a widely applied model to analyze general and specific abilities. Extensions of bifactor models additionally include criterion variables. In such extended bifactor models, the general and specific factors can be correlated with criterion variables. Moreover, the influence of general and specific factors on criterion variables can be scrutinized in latent multiple regression models that are built on bifactor measurement models. This study employs an extended bifactor model to predict mathematics and English grades by three facets of intelligence (number series, verbal analogies, and unfolding). We show that, if the observed variables do not differ in their loadings, extended bifactor models are not identified and not applicable. Moreover, we reveal that standard errors of regression weights in extended bifactor models can be very large and, thus, lead to invalid conclusions. A formal proof of the nonidentification is presented. Subsequently, we suggest alternative approaches for predicting criterion variables by general and specific factors. In particular, we illustrate how (1) composite ability factors can be defined in extended first-order factor models and (2) how bifactor(*S*-1) models can be applied. The differences between first-order factor models and bifactor(*S*-1) models for predicting criterion variables are discussed in detail and illustrated with the empirical example.

## 1. Introduction

In 1904, Charles Spearman [1] published his groundbreaking article “*General intelligence objectively determined and measured*” that has been affecting intelligence research since then. In this paper Spearman stated that “all branches of intellectual activity have in common one fundamental function (or groups of functions), whereas the remaining or specific elements of the activity seem in every case to be wholly different from that in all the others” (p. 284). Given Spearman’s distinction into general and specific cognitive abilities, one fundamental topic of intelligence research has been the question to which degree these general and specific facets are important for predicting real-world criteria (e.g., [2,3]; for an overview see [4]). In other words, is it sufficient to consider *g* alone or do the other specific factors (also sometimes referred to as narrower factors) contribute in an essential way?

Around the year 2000, there was a unanimously agreed answer to this question. Several authors concluded that specific abilities do not explain much variance beyond *g* (e.g., [5,6]). In the past decade, however, this consensus has shifted from “not much more than *g*” (see [7]) to the notion that there may be something more than *g* predicting real-world criteria. Reflecting this shift, Kell and Lang [4] summarize that “recent studies have variously demonstrated the importance of narrower abilities above and beyond *g*.” (p. 11). However, this debate is far from settled [8].

An apparent issue in evaluating discrepant findings across studies is the statistical approach applied. Much of the earlier evidence was based on hierarchical regression analyses, in which *g* (the first unrotated principle component) was entered in the first and specific cognitive abilities in the second step (e.g., [6]). Other studies relied on relative importance analysis (e.g., [9]), mediation models, in which criteria are predicted by *g* which in turn is predicted by specific abilities (e.g., [10]), as well as meta-analytical procedures (e.g., [11,12]). There is another prominent approach to separate general from specific abilities: the bifactor model [13]. Although its introduction dates way back, the bifactor model is recently and increasingly applied in studies predicting criterion variables by general and specific factors, not only in the area of cognitive abilities and school performance measures (e.g., [14,15,16,17,18,19,20,21,22,23,24]), but also in different other areas of psychological research such as motivation and engagement (e.g., [25,26,27]), clinical psychology (e.g., [28,29,30]), organizational psychology (e.g., [31]), personality psychology (e.g., [32,33]), and media psychology (e.g., [34]). The multitude of recently published studies using the bifactor model shows that it has become a standard model for predicting criterion variables by general and specific components.

In the current study, we seek to contribute to the debate on general versus specific cognitive abilities as predictors of real-life criteria by taking a closer look at the bifactor model. We will describe the basic idea of the bifactor model and its applicability for predicting criterion variables. We will also apply it to the data set provided by the editors of this special issue. In particular, we will show that the bifactor model is not generally identified when the prediction of criterion variables comes into play and can be affected by estimation problems such as large standard errors of regression weights. To our knowledge, this insight has not been published previously. Subsequently, we will illustrate and discuss alternatives to the bifactor model. First, we will present a first-order factor model with correlated factors as well as an extension of this model, in which a composite intelligence factor is defined by the best linear combination of facets for predicting criterion variables. Second, we will discuss bifactor(*S*-1) models, which constitute recently developed alternatives to the bifactor approach [35]. We conclude that bifactor(*S*-1) models might be more appropriate for predicting criterion variables by general and specific factors in certain research areas.

### Bifactor Model

**Figure 1:**
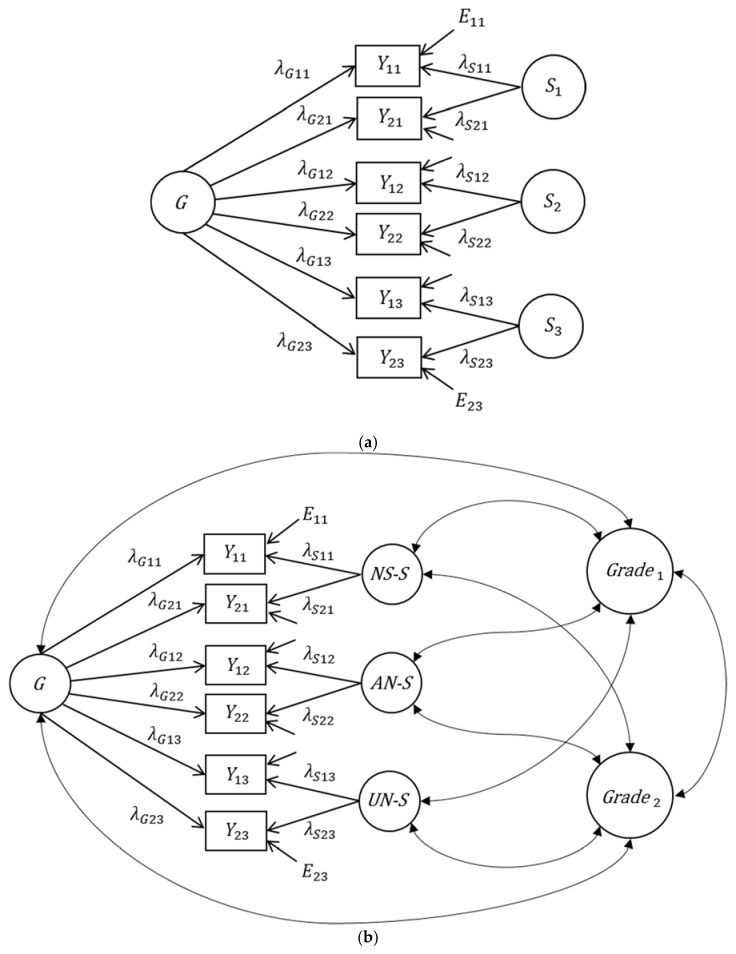

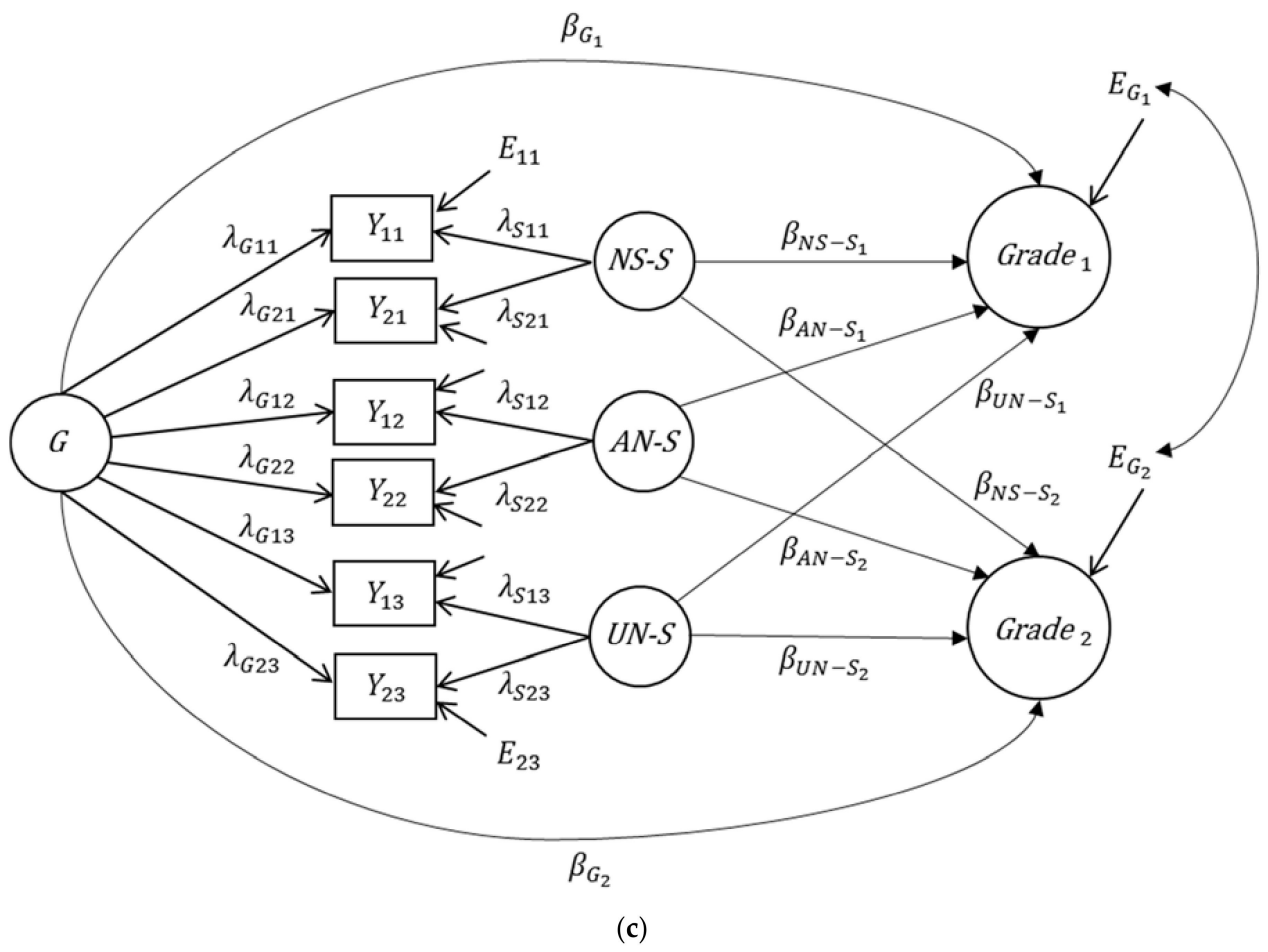
Bifactor model and its extensions to criterion variables. (**a**) Bifactor model without criterion variables, (**b**) bifactor model with correlating criterion variables (grades), and (**c**) multiple latent regression bifactor model. The factors of the extended models depicted refer to the empirical application. *G*: general factor, *S_k_*: specific factors; *NS-S*: specific factor number series, *AN-S*: specific factor verbal analogies, *UN-S*: specific factor unfolding. *E_ik_*: measurement error variables, *E*_G1_/*E*_G2_: residuals, *λ*: loading parameters, *β*: regression coefficients, *i*: indicator, *k*: facet.

The bifactor model was introduced by Holzinger and Swineford [13] to separate general from specific factors in the measurement of cognitive abilities. Although this model is quite old, it was seldom applied in the first seventy years of its existence. It has only become a standard for modeling *g*-factor structures in the last ten years [32,35,36,37]. When this model is applied to measure general and specific cognitive abilities, *g* is represented by a general factor that is common to all cognitive ability tests included in a study (see Figure 1a). In case of the three cognitive abilities considered in this study (number series, verbal analogies, and unfolding), the general factor represents variance that is shared by all three abilities. The cognitive ability tests additionally load on separate orthogonal factors—the specific factors. So, each specific factor, also sometimes referred to as group factor (e.g., [37]), represents a unique narrow ability. Because all factors in the classical bifactor model are assumed to be uncorrelated, the variance of an observed measure of cognitive abilities can be decomposed into three parts: (1) measurement error, (2) the general factor, and (3) the specific factors. This decomposition of variance allows estimating to which degree observed differences in cognitive abilities are determined by *g* or by the specific components.

The bifactor model is also considered a very attractive model for predicting criterion variables by general and specific factors (e.g., [32]). It becomes attractive for such purposes since the general and the specific factors—as specified in the bifactor model—are uncorrelated, thus representing unique variance that is not shared with the other factors. Hence, they contribute independently of each other to the prediction of the criterion variable. In other words, the regression coefficients in a multiple regression analysis (see Figure 1c) do not depend on the other factors in the model. Consequently, the explained criterion variance can be additively decomposed into components that are determined by each general and specific factor.

On the one hand, these properties make the bifactor model very attractive for applied researchers. On the other hand, many studies that used bifactor models to predict criterion variables, hereinafter referred to as extended bifactor models (see Figure 1c), showed results that were not theoretically expected. For example, some of these studies revealed loadings (of indicators either on the *g* factor or on the specific factors) that were insignificant or even negative—although these items were theoretically assumed as indicators of these factors (e.g., [19,25,27,28,29,30]). Moreover, it was often observed that one of the specific factors was not necessary to predict criterion variables by general and specific factors (e.g., [14,18,19,32,33]). Similar results were often found in applications of non-extended versions of the bifactor model (see [35], for an extensive discussion of application problems of the bifactor model).

Beyond the unexpected results found in several studies that used bifactor models, its applicability is affected by a more fundamental problem. When a bifactor model is extended to criterion variables, the model is not globally identified—although the model without criterion variables is. As we will show below, the extended bifactor model is not applicable if the indicators do not differ in their loadings: it might be affected by estimation problems (e.g., large standard errors of regression coefficients) or even be unidentified. Next, we will use the data set provided by the editors of the special issue to illustrate this problem.

## 2. Description of the Empirical Study

### 2.1. Participants and Materials

We analyzed the data set provided by Kell and Lang [38]. It includes data from *n* = 219 individuals. Gender was almost equally distributed among the sample (53% female). Their mean age was 16 years (*SD* = 1.49, range = 13 to 23).

The data set included three subtests of the Wilde Intelligence Test 2 [39]. These subtests were: verbal analogies (complete a word pair so that it logically matches a given other word pair), number series (find the logical next number in a series of numbers), and figural unfolding (identify the 3-dimensional form that can be created by a given two-dimensional folding sheet). The number of correctly solved items within the time limit of each subtest serves as a participant’s score. For the purpose of the current paper, we conducted an odd-even split of subtest items to obtain two indicators per each subtest. If achievement tests are split into two parts, an odd-even split is recommended for two main reasons. First, such tests usually contain a time limit. Hence, splitting tests in other ways would result in unbalanced parcels (one parcel would contain “later” items for which the time limit might have been more of a concern). Second, items are usually ordered so that item difficulty increases. Hence, the odd-even split ensures that items with approximately equal difficulty are assigned to both parcels.

We used two of the grades provided in the data set, mathematics and English. We chose these grades because we wanted to include a numerical and a verbal criterion. For more details about the data set and its collection, see Kell and Lang [38].

### 2.2. Data Analysis

The data was analyzed using the computer program Mplus Version 8 [40]. The observed intelligence test scores were taken as continuous variables whereas the grades were defined as categorical variables with ordered categories. The estimator used was the WLSMV estimator which is recommended for this type of analysis [40]. The correlations between the grades are polychoric correlations, the correlations between the grades and the intelligence variables are polyserial correlations whereas the correlations between the intelligence variables are Pearson correlations. The correlation matrix of the observed variables, on which the analyses are based, is given in Table 1. The correlations between test halves (created by an odd-even split) of the same intelligence facets were relatively large (between *r* = 0.687 and *r* = 0.787), thus showing that it is reasonable to consider the respective halves as indicators of the same latent intelligence factor. Correlations between grades and observed intelligence variables ranged from *r* = 0.097 to *r* = 0.378. The correlation between the two grades were *r* = 0.469.

### 2.3. Application of the Bifactor Model

In a first step, we analyzed a bifactor model with equal loadings (loadings of 1) on the general and specific factors. All factors were allowed to correlate with the two criterion variables (see Figure 1b). The estimation of this model did not converge—although a bifactor model with equal loadings but without the two criterion variables fitted the data very well (*χ*^2^ = 10.121, *df* = 11, *p* = 0.520). These estimation problems are due to the fact that a bifactor model with equal loadings and covariates is not identified (i.e., it is not possible to get a unique solution for the parameter estimates). Their nonidentifiability can be explained as follows: In a bifactor model with equal loadings, the covariance of an observed indicator of intelligence and a criterion variable is additively decomposed into (a) the covariance of the criterion variable with the *g* factor and (b) the variance of the criterion variable with a specific factor. Next, a formal proof is presented.

In the model with equal factor loadings, an observed variable *Y_ik_* is decomposed in the following way (the first index *i* refers to the indicator, the second indicator *k* to the facet):

$$\;Y_{ik}=G+S_{k}+E_{ik}\;$$

Assuming that the error variables $E_{ik}$ are uncorrelated with the criterion variables, the covariance of the observed variables *Y_ik_* and a criterion variable *C* can be decomposed in the following way:

$$\;Cov(Y_{ik},C)=Cov\left(G+S_{k}+E_{ik},\;C\right)=Cov\left(G,C\right)+Cov\left(S_{k},C\right)\;$$

The covariance $Cov(Y_{ik},C)$ can be easily estimated by the sample covariance. However, because each covariance $Cov(Y_{ik},C)$ is additively decomposed in essentially the same two components, there is no unique solution to estimate Cov(G,C) independently from $Cov\left(S_{k},C\right)$. Hence, the model is not identified.

The decomposition of the covariance $Cov(Y_{ik},C)$ holds for all indicators of intelligence and all specific factors. According to this decomposition there is an infinite number of combinations of Cov(G,C) and $Cov\left(S_{k},C\right)$. While this formal proof is herein only presented for the covariance of $Cov(Y_{ik},C)$, it also applies to polyserial correlations considered in the empirical application. In case of polyserial correlations, the variable *C* refers to the continuous variable that is underlying the observed categorical variable. 

The nonidentification of the bifactor model with equal loadings has an important implication for the general research question of whether *g* factor versus specific factors predict criterion variables. That is, the model can only be identified and the estimation problems only be solved if one fixes one of the covariances to 0, i.e., either Cov(G,C)=0 or $Cov\left(S_{k},C\right)=0$. When we fixed $Cov\left(S_{k},C\right)=0$ for all three specific factors of our model, the model was identified and fitted the data very well (*χ*^2^ = 17.862, *df* = 21, *p* = 0.658). In this model, the *g* factor was significantly correlated with the mathematics grades (*r* = 0.574) and the English grades (*r* = 0.344). Consequently, one would conclude that only *g* is necessary for predicting grades. However, when we fixed Cov(G,C)=0, the respective model was also identified and fitted the data very well (*χ*^2^ = 14.373, *df* = 17, *p* = 0.641). In this model, the *g* factor was not correlated with the grades; instead all the specific factors were significantly correlated with the mathematics and the English grades (mathematics—*NS*: *r* = 0.519, *AN*: *r* = 0.572, *UN*: *r* = 0.452; English—*NS*: *r* = 0.319, *AN*: *r* = 0.434, *UN*: *r* = 0.184). Hence, this analysis led to exactly the opposite conclusion: The *g* factor is irrelevant for predicting grades, only specific factors are relevant. It is important to note that both conclusions are arbitrary, and that the model with equal loadings is in no way suitable for analyzing this research question.

The identification of models with freely estimated loadings on the general and specific factors is more complex and depends on the number of indicators and specific factors. If loadings on the *g* factor are not fixed to be equal, the model with correlating criterion variables (see Figure 1b) is identified (see Appendix A for a more formal discussion of this issue). However, because there are only two indicators for each specific factor, their loadings have to be fixed to 1. The corresponding model fitted the data very well (*χ*^2^ = 8.318, *df* = 10, *p* = 0.598). The estimated parameters of this model are presented in Table 2^1^. All estimated *g* factor loadings were very high. The correlations of the mathematics grades with the *g* factor and with the specific factors were similar, but not significantly different from 0. For the English grades, the correlations differed more: The specific factor of verbal analogies showed the highest correlation with the English grades. However, the correlations were also not significantly different from 0. The results showed that neither the *g* factor nor the specific factors were correlated with the grades. According to these results, cognitive ability would not be a predictor of grades—which would be in contrast to ample research (e.g., [41]). However, it is important to note that the standard errors for the covariances between the factors and the grades were very high, meaning that they were imprecisely estimated. After fixing the correlations between the specific factors and the grades to 0, the model fitted the data very well (*χ*^2^ = 16.998, *df* = 16, *p* = 0.386). In this model, the standard errors for the estimated covariances between the *g* factor and the grades were much smaller (mathematics: 0.128, English: 0.18). As a result, the *g* factor was significantly correlated with both grades (mathematics: *r* = 0.568, English: *r* = 0.341). So, in this analysis, *g* showed strong correlations with the grades whereas the specific factors were irrelevant. However, fixing the correlations of *g* with the grades to 0 and letting the specific factors correlate with the grades, resulted in the very opposite conclusion. Again, this model showed a very good fit (*χ*^2^ = 8.185, *df* = 12, *p* = 0.771) and the standard errors of the covariances between the specific factors and the grades were lower (between 0.126 and 0.136). This time, however, all specific factors were significantly correlated with all grades (Mathematics—*NS*: *r* = 0.570, *AN*: *r* = 0.522, *UN*: *r* = 0.450; English—*NS*: *r* = 0.350, *AN*: *r* = 0.396, *UN*: *r* = 0.183). While all specific factors were relevant, in this case the *g* factor was irrelevant for predicting individual differences in school grades.

We observed the same problem in a multiple regression analysis in which the grades were regressed on the general and specific factors (see Figure 1c). In this model—which yielded the same fit as the model with all correlations—all regression coefficients showed high standard errors and were not significantly different from 0 (see Table 3). Fixing the regression coefficients on all specific factors to 0 led to a fitting model with significant regression coefficients for the *g* factor, whereas fixing the regression coefficients on the *g* factor to 0 resulted in a fitting model with significant regression weights for the specific factors (with exception of the unfolding factor for the English grades). It is important to note that in the multiple regression analysis the *g* factor and the specific factors were uncorrelated. Therefore, the high standard errors in this model cannot be due to multicollinearity. Instead, it shows that there are more fundamental application problems of the bifactor model for predicting criterion variables.

## 3. Alternatives to Extended Bifactor Models

Because the application of bifactor models for predicting criterion variables by facets of intelligence might lead to invalid conclusions, alternative models might be more appropriate for predicting criterion variables by facets of intelligence. We will discuss two alternative approaches. First, we will illustrate the application of an extended first-order factor model and then of an extended bifactor(*S*-1) model.

### 3.1. Application of the Extended First-Order Factor Model

**Figure 2:**
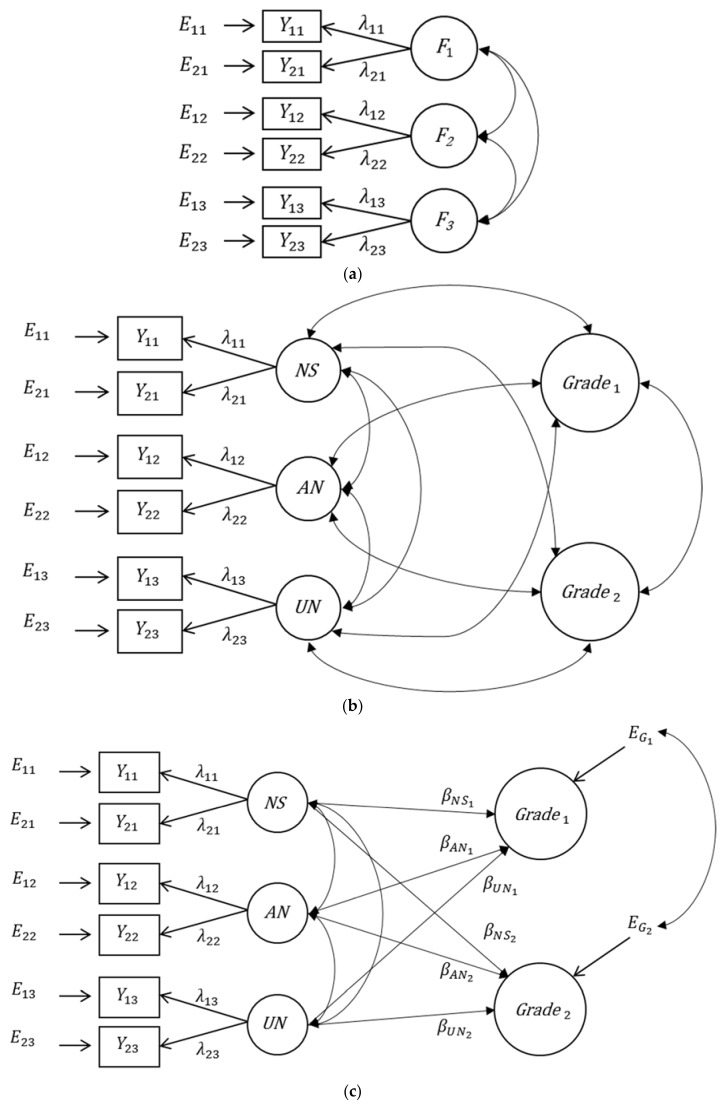

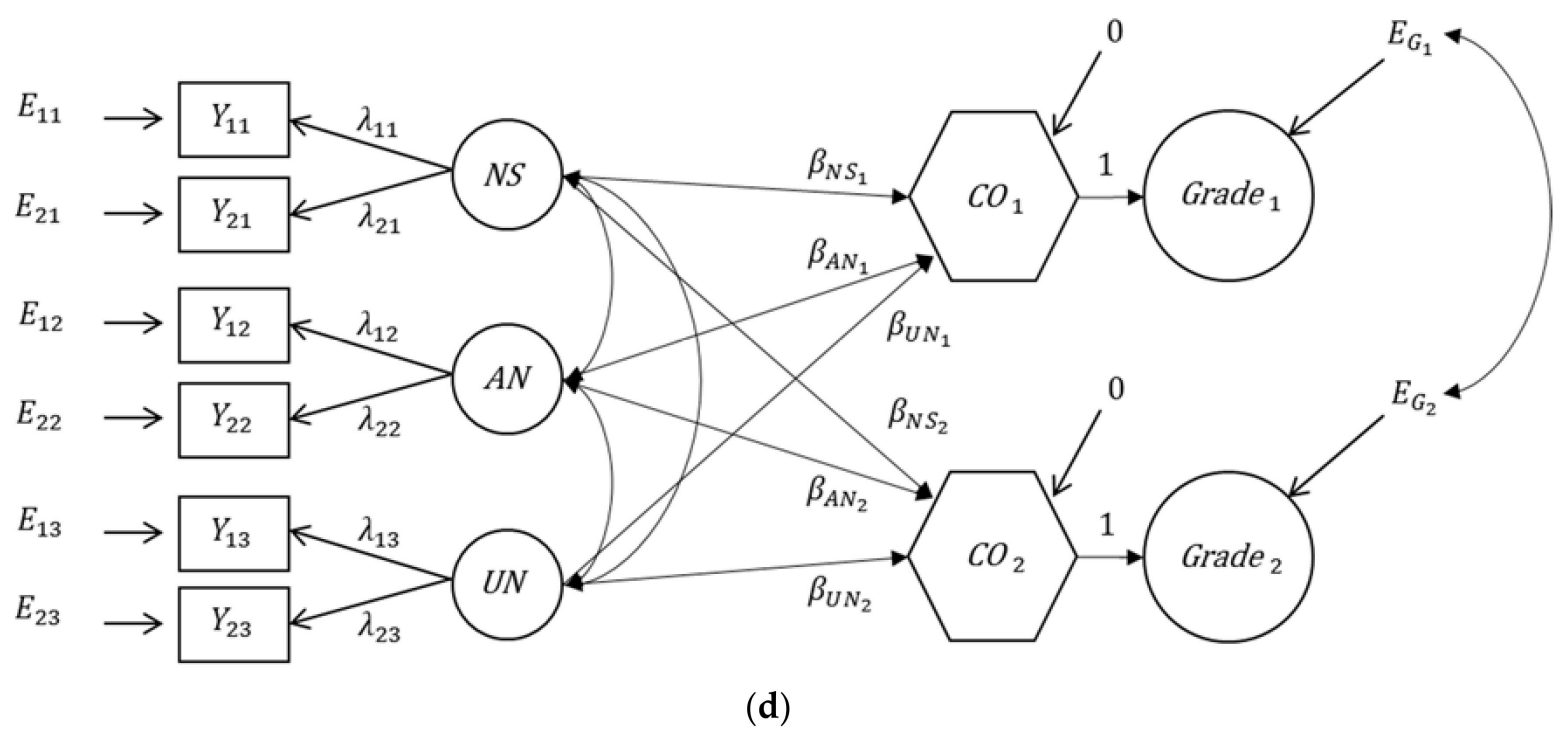
Modell with correlated first-order factors. (**a**) Model without criterion variables, (**b**) model with correlating criterion variables, (**c**) multiple latent regression model, and (**d**) multiple latent regression model with composite factors. *F_k_*: facet factors, *E_ik_*: measurement error variables, *NS*: facet factor number series, *AN*: facet factor verbal analogies, *UN*: facet factor unfolding, *CO*_1_*/CO*_2_: composite factors, *E*_G1_/*E*_G2_: residuals *λ*: loading parameters, *β*: regression coefficients, *i*: indicator, *k*: facet.

In the first-order factor model there is a common factor for all indicators belonging to the same facet of a construct (see Figure 2a). The factors are correlated; the correlations show how distinct or comparable the different facets are. It is a very general model as the correlations of the latent factors are not restricted in any way (e.g., by a common general factor) and it allows us to test whether the facets can be clearly separated in the intended way (e.g., without cross-loadings). An extension of this model to criterion variables is shown in Figure 2b. We applied this model to estimate the correlations between the intelligence facet factors and the grades. Because the two indicators were created through an odd-even split, we assumed that the loadings of the indicators on the factors did not differ between the two indicators. For identification reasons, the default Mplus settings were applied, meaning that the unstandardized factor loadings were fixed to 1 and the mean values of the factors were fixed to 0.

This model fitted the data very well (*χ*^2^ = 13.929, *df* = 15, *p* = 0.531) and did not fit significantly worse than a model with unrestricted loadings (*χ*^2^ = 9.308, *df* = 12, *p* = 0.676; scaled *χ*^2^-difference = 2.933, *df* = 3, *p* = 0.402). The results of this analysis are presented in Table 4. The standardized factor loadings and therefore also the reliabilities of the observed indicators were sufficiently high for all observed variables. The correlations between the three facet factors were relatively similar and ranged from *r* = 0.408 to *r* = 0.464. Hence, the facets were sufficiently distinct to consider them as different facets of intelligence. The correlations of the factors with the mathematics grades were all significantly different from 0 and ranged from *r* = 0.349 (unfolding) to *r* = 0.400 (verbal analogies) showing that they differed only slightly between the intelligence facets. The correlations with the English grades were also significantly different from 0, but they differed more strongly between the facets. The strongest correlation of *r* = 0.304 was found for verbal analogies, the correlations with the facets number series and unfolding were *r* = 0.242 and *r* = 0.142, respectively.

The model can be easily extended to predict criterion variables. Figure 2c depicts a multiple regression model with two criterion variables (the two grades in the study presented). The regression coefficients in this model have the same meaning as in a multiple regression analysis. They indicate to which degree a facet of a multidimensional construct contributes to predicting the criterion variable beyond all other facets included in the model. If the regression coefficient of a facet factor is not significantly different from 0, this indicates that this facet is not an important addition to the other facets in predicting the criterion variable. The residuals of the two criterion variables can be correlated. This partial correlation indicates that part of the correlation of the criterion variables that is not due to the common predictor variables. Table 5 shows that the regression coefficients differ between the two grades. Verbal analogies were the strongest predictor of both grades; it predicted both grades almost identically well. The two other intelligence facets had also significant regression weights for the mathematics grades, but their regression weights were small and not significantly different from 0 for the English grades. Consequently, the explained variance also differed between the two grades. Whereas 23.3 percent of the variance of the mathematics grades was explained by the three intelligence facets together, only 10.6 percent of the variance of the English grades was predictable by the three intelligence facets. The residual correlation of *r* = 0.390 indicated that the association of the two grades cannot be perfectly predicted by the three facets of intelligence.

Notably, the multiple regression model can be formulated in a slightly different but equivalent way: A latent composite variable can be introduced reflecting the linear combination of the facet factors for predicting a criterion variable [42]; this model is shown in Figure 2d. In this figure, we use a hexagon to represent a composite variable, an exact linear function of the three composite indicators [43]. The values of this composite variable are the values of the criterion variable predicted by the facet factors. They correspond to the predicted values $\hat{y}$ of a dependent variably *Y* in a multiple regression analysis. A composite variable combines the information in the single intelligence facets in such a way that all aspects that are relevant for predicting the criterion variable are represented by this composite factor. Consequently, the single facet factors do not contribute to predicting the criterion variable beyond this composite factor. Their contribution is represented by their regression weight determining the composite factor. While this composite factor is not generally necessary for predicting the criterion variables, it might be particularly important in some specific cases. In personnel assessment, for example, one wants to select those individuals whose intelligence scores might best fit the requirements of a vacant position. The composite score may be built to best reflect these specific requirements (if appropriate criterion-related validity studies are available). The composite score thus represents an intelligence score of this person, specifically tailored to the assessment purpose. We argue that—if appropriate evidence allows for it—composite scores that are tailored to the purpose at hand can be more appropriate than aggregating intelligence facets according to their loadings on broader factors (e.g., on the first principal component of all observed intelligence measures or on a *g* factor in a bifactor model). In fact, understanding a broader measure of intelligence as the best combination of intelligence facets is in line with modern approaches of validity [44,45,46,47]. According to these approaches, validity is not a property of a psychological test. Rather, a psychometric test can be applied for different purposes (here: predicting different grades) and the information has to be combined and interpreted in the most appropriate way to arrive at valid conclusions. Therefore, it might not always be reasonable to rely on *g* as an underlying variable (“property of a test”) such as in a bifactor model, but to look for the best combination of test scores for a specific purpose. Thus, also from a validity-related point-of-view, the bifactor model might be—independently from the estimation problems we have described—a less optimal model.

### 3.2. Application of the Bifactor(S-1) Model

**Figure 3:**
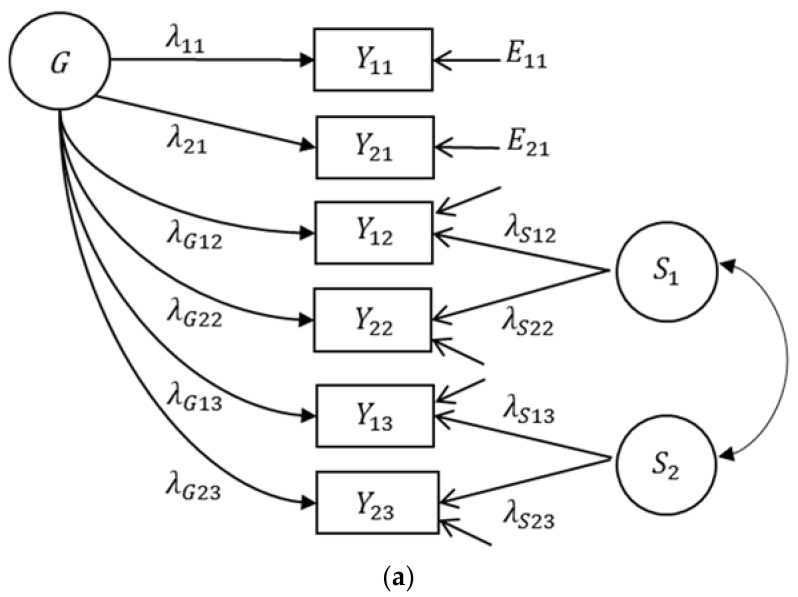

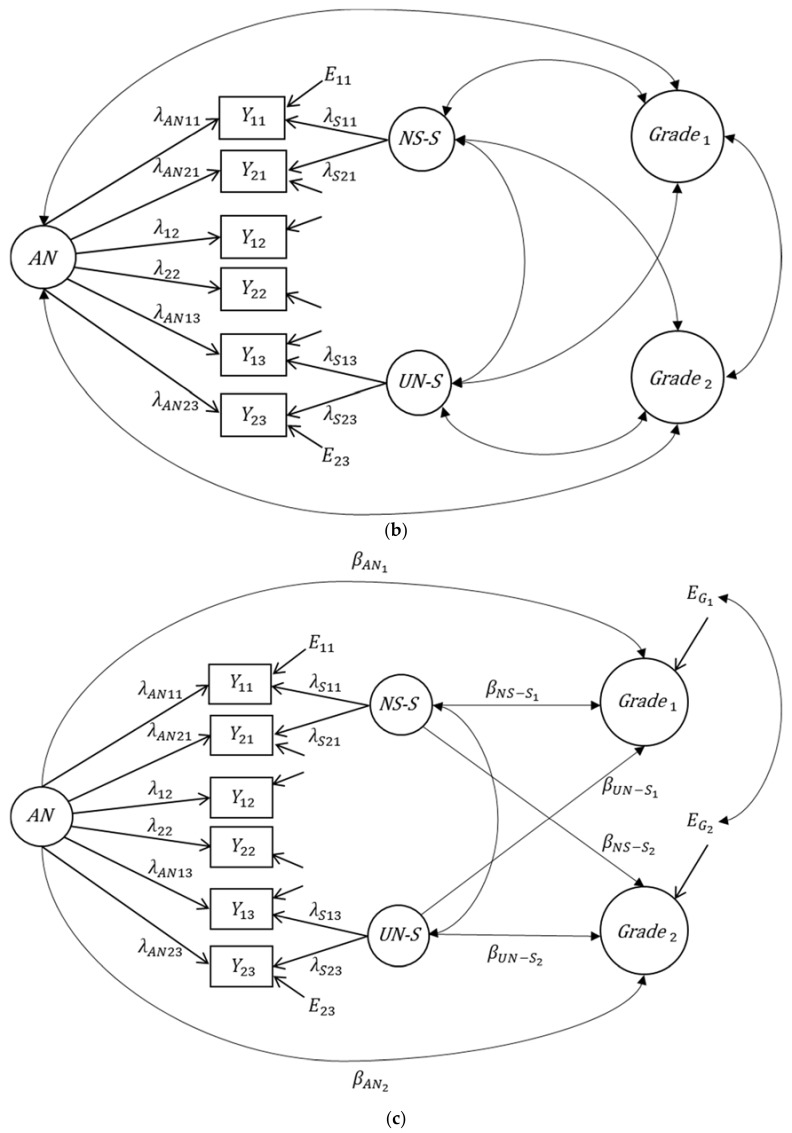
Bifactor(*S*-1) model and its extensions to criterion variables. (**a**) Bifactor(*S*-1) model without criterion variables, (**b**) bifactor(*S*-1) model with correlating criterion variables (grades), and (**c**) multiple latent regression bifactor(*S*-1) model. The factors of the extended models depicted refer to the empirical application. *G*: general factor, *S_k_*: specific factors; *NS-S*: specific factor number series, *AN-S*: specific factor verbal analogies, *UN-S*: specific factor unfolding. *E_ik_*: measurement error variables, *E*_G1_/*E*_G2_: residuals, *λ*: loading parameters, *β*: regression coefficients, *i*: indicator, *k*: facet.

A bifactor(*S*-1) model is a variant of a bifactor model in which one specific factor is omitted (see Figure 3a). In this model the *g* factor represents individual differences on the facet that is theoretically selected as the reference facet. Therefore, it is not a general factor as it is assumed in a traditional *g* factor model. Rather, it is intelligence as captured by the reference facet. A specific factor represents that part of a facet that cannot be predicted by the reference facet. Unlike the classical bifactor model, the specific factors in the bifactor(*S*-1) model can be correlated. This partial correlation indicates whether two facets have something in common that is not shared with the reference facet. A bifactor(*S*-1) can be defined in such a way that it is a reformulation of the model with correlated first-order factors (see Figure 2a) and shows the same fit [48]. Because first-order factor models usually do not show anomalous results, the bifactor(*S*-1) model is usually also not affected by the estimation problems found in many applications of the bifactor model [35]. Applying a bifactor(*S*-1) model may also be a better alternative to bifactor models when it comes to predicting real-world criteria (see Figure 3b,c), because this model avoids the identification and estimation problems inherent in the extended bifactor model.

Several researchers have applied the bifactor(*S*-1) model for predicting criterion variables by cognitive abilities. This was the case even in one of the very early applications of bifactor models of intelligence to predict achievement in different school subjects [49]. In their application of a bifactor(*S*-1) model, Holzinger and Swineford [49] defined the *g* factor by three reference tests (without indicating a specific factor) and a specific factor by eight tests having loadings on the *g* factor as well as on a specific spatial ability factor.^2^ Also Gustafsson and Balke [2] selected one indicator (letter grouping) to define the *g* factor of aptitudes. Other examples of applying bifactor(*S*-1) models are Brunner’s [17] and Saß et al.’s [21] studies, in which a *g* factor of cognitive abilities was defined by fluid ability. Likewise, Benson et al. [15] defined their *g* factor of cognitive abilities by the test story completion. Notably, many applications of the standard bifactor model are essentially bifactor(*S*-1) models, because often one of the specific factors in the standard bifactor model does not have substantive variance (see [35]). In such cases, the specific factor without substantive variance becomes the reference facet and defines the meaning of the *g* factor. Unfortunately, this is very rarely stated explicitly in such cases. In bifactor(*S*-1) models, on the contrary, the *g* factor is theoretically and explicitly defined by a reference facet, i.e., the meaning of *g* depends on the choice of the reference facet. Thus, another advantage of the bifactor(*S*-1) model is that the researcher explicitly determines the meaning of the reference facet factor and communicates it. Moreover, it avoids estimation problems that are related to overfactorization (i.e., specifying a factor that has no variance). 

In the bifactor(*S*-1) model, the regression coefficients for predicting criterion variables by facets of intelligence have a special meaning. We will discuss their meaning by referring to the empirical example presented. For applying the bifactor(*S*-1) model, one facet has to be chosen as the reference facet. In the current analyses, we chose the facet verbal analogies as the reference facet, because it was most strongly correlated with both grades. However, the reference facet can also be selected on a theoretical basis. The bifactor(*S*-1) model then tested whether the remaining facets contribute to the prediction of grades above and beyond the reference facet. Because the first-order model showed that the indicators did not differ in their factor loadings, we also assumed that the indicators of a facet showed equal factor loadings in the bifactor(*S*-1) model. 

The fit of the bifactor(*S*-1) model with the two grades as correlated criterion variables (see Figure 2a) was equivalent to the first-order factor model (*χ*^2^ = 13.929, *df* = 15, *p* = 0.531). This result reflects that both models are simply reformulations of each other. In addition, the correlations between the reference facet and the two grades did not differ from the correlations that were observed in the first-order model. This shows that the meaning of the reference facet does not change from one model to the other. There is, however, an important difference between both models. In the bifactor(*S*-1) model, the non-reference factors are residualized with respect to the reference facet. Consequently, the meaning of the non-reference facets and their correlations with the criterion variables change. Specifically, the correlations between the specific factors of the bifactor(*S*-1) model and the grades indicate whether the non-reference factors contain variance that is not shared with the reference facet, but that is shared with the grades. The correlations between the specific factors of the bifactor(*S*-1) model and the grades are part (semi-partial) correlations (i.e., correlations between the grades, on the one hand side, and the non-reference facets that are residualized with respect to the reference facet, on the other hand side). 

The estimated parameters of the bifactor(*S*-1) model when applied to the empirical example are presented in Table 6. All observed intelligence variables showed substantive loadings on the common factor (i.e., verbal analogies reference facet factor). The standardized loadings of the observed verbal analogies indicators were identical to those obtained from the first-order factor model (because the reference facet factor is identical to the first-order factor verbal analogies). The standardized factor loadings of the non-reference factor indicators were smaller (between 0.332 and 0.412); they can be interpreted as correlations between the indicators of the other non-reference facets (i.e., number series and unfolding) and the common verbal analogies factor (i.e., reference facet). The standardized loadings pertaining to the specific factors were higher (between 0.744 and 0.787) showing that the non-reference facets indicators assessed a specific part of these facets that was not shared with the common verbal reasoning factor. The common verbal reasoning factor was strongly correlated with the mathematics grades (*r* = 0.400) and the English grades (*r* = 0.304). Significant correlations were obtained between the specific factors and the mathematics grades (*r* = 0.203 and *r* = 0.235), but not between the specific factors and the English grades. Hence, number series and unfolding were not important for understanding individual differences in English grades, if individual differences in verbal analogies were controlled for.

An extension of the bifactor(*S*-1) model to a multiple regression model is depicted in Figure 3c. The estimated parameters are presented in Table 7. For mathematics grades, the results show that the specific factors have a predictive power above and beyond the common verbal analogies reference factor. This was not the case for English grades. The differences between the bifactor(*S*-1) regression model and the first-order factor regression model can be illustrated by comparing the unstandardized regression coefficients in Table 3 and Table 7. They only differ for verbal analogies, the facet taken as reference in the bifactor(*S*-1) model. Whereas in the first-order factor model, the regression coefficient of the verbal analogies facet indicates its predictive power above and beyond the two other facets, its regression coefficient in the bifactor(*S*-1) model equals the regression coefficient in a simple regression model (because it is not corrected for its correlation with the remaining non-reference facets). Therefore, in the first-order factor model, the regression coefficient of verbal analogies depends on the other facets considered. If other facets were added to the model, this would affect the regression coefficient of verbal analogies (assuming that the added facets are correlated with verbal analogies). Hence, in order to compare the influence of verbal analogies on the grades across different studies, it is always necessary to take all other included facets into consideration. In the bifactor(*S*-1) model, however, the regression coefficient of verbal analogies, the reference facet, does not depend on other facets. Adding other facets of intelligence would not change the regression coefficient of verbal analogies. As a result, the regression coefficient of verbal analogies for predicting the same criterion variables can be compared across different studies without considering all other facets. 

It is important to note that the correlations and the regression coefficients in the bifactor(*S*-1) model can change if one selects another facet as the reference facet. When we changed the reference facet in our empirical example, however, neither the fit of the bifactor(*S*-1) model nor did the explained variance in the criterion variables changed. When we used number series as reference facet, for example, the regression coefficient of verbal analogies—now considered a specific facet—significantly predicted English grades, in addition to the reference facet (see Table 8). When predicting mathematics grades, the specific factors of verbal analogies and unfolding had an additional effect. Note that the choice of the reference facet depends on the research question and can also differ between criterion variables (e.g., verbal analogies might be chosen as reference facet for language grades and number series as reference facet for mathematics and science grades). 

## 4. Discussion

The bifactor model has become a standard model for analyzing general and specific factors [35,37]. One major advantage of the bifactor model is that all factors are uncorrelated. If one extends the model to a multiple regression framework and uses this model to predict criterion variables by general and specific factors, then the general and specific factors are independent sources of prediction. So, the problem of multicollinearity is avoided. Hence, the regression weights indicate to which degree general and specific abilities are important for predicting criterion variables. However, our empirical application revealed severe identification and estimation problems which strongly limit the applicability of the bifactor model for predicting criterion variables. First, the bifactor model with criterion variables as covariates is not identified if (a) the indicators do not differ in their loadings on the general and specific factors, and (b) both the general and specific factors are correlated with the criterion variables. In the herein conducted empirical application of the bifactor model, the indicators did not differ significantly in their loadings. Therefore, the extended bifactor model with equal loadings could not be applied. Equal loadings might be rather common in intelligence research, because many authors of intelligence tests might base their item selection on the Rasch model [50], also called the one-parameter logistic model. The Rasch model has many advantages such as specific objectivity, the fact that item parameters can be independently estimated from person parameters and that the total score is a sufficient statistic for the ability parameter. Particularly, applications of bifactor models on item parcels or items that do not differ in their discrimination—as is the case in the one-parameter logistic model—will result in identification problems. The same is true for tests developed on the basis of the classical test theory, where equal factor loadings are desirable for test authors (mostly because of the ubiquitous use of Cronbach’s alpha, which is only a measure of test score reliability if the items do not differ in their loadings). Hence, applying well-constructed tests in research on intelligence might often result in a situation where the loadings are equal or similar. 

However, in the case of equal loadings, the extended bifactor model is only identified if the correlations (or regression weights) of either the general factor with the criterion variables or of the specific factors with the criterion variables are fixed to 0. This has a serious implication for research on general vs. specific factors predicting real-world criteria: The bifactor model is not suitable for deciding whether the general or the specific factors are more important for predicting criterion variables. As we have shown in the empirical application, one can specify the model in such a way that either the *g* factor or the specific factors are the relevant source of individual differences in the criterion variables, thereby making this model arbitrary for determining the relative importance of *g* versus specific abilities. In order to get an identified bifactor model, we had to freely estimate the factor loadings of the general factor. However, even for this (then identified) model, the standard errors of the correlation and regression coefficients were so large that none of the coefficients were significant—although generally strong associations between intelligence facets and school grades existed. Hence, applying the bifactor model with criterion (or other) variables as covariates can result in invalid conclusions about the importance of general and specific factors. 

It is important to note that the high standard errors are not due to multicollinearity, but seem to be a property of the model itself, as the estimated factor loadings were close to the situation of non-identification (i.e., almost equal). Fixing either the correlations between the grades and the general factor or between the grades and the specific factors results in lower standard errors and significant correlations and regression weights. Again, however, it cannot be appropriately decided whether the general factor or the specific factors are the relevant source of individual differences. This fact even offers some possibilities for misuse. For example, proponents of the *g* factor might report the fit coefficients of the model with all correlation coefficients estimated and with the correlation coefficients of the specific factors fixed to zero. They might argue (and statistically test) that the two models fit equally well and, therefore, report only the results of the reduced model showing significant *g* factor correlations. This would lead to the conclusion that the specific factors are irrelevant for predicting criterion variables. Conversely, proponents of specific factors might apply the same strategy and use the same arguments to show that *g* is irrelevant (e.g., only measuring response styles) and only the specific factors are relevant. According to our analyses, both conclusions are arbitrary and not valid. Because of this arbitrariness, the question arises what the general factor and the specific factors mean. 

Because of the strong limitations of the extended bifactor model, we proposed two alternative approaches. The first alternative is an extension of the first-order factor model to a latent multiple regression model in which the criterion variables are regressed on different facet factors. The regression weights in such a model reflect the impact of a facet on a criterion variable, after controlling for all other facets. This is equivalent to residualizing a facet with respect to all other facets and removing that part of a facet that is already shared with all remaining facets in the model. Thus, a regression weight of 0 means that the facet does not contribute to the prediction of the criterion variable above and beyond all other facets in the model. When applied to general and specific abilities, we have shown that the multiple regression model can be formulated in such a way that a composite factor is defined as the best linear combination of different facets. The importance of a specific facet is represented by the weight with which the specific facet contributes to the composite factor. Because of the properties of the multiple regression models, the meaning of the composite factor can differ between different criterion variables. That means that depending on the purpose of a study, the composite factor always represents the best possible combination of the information (specific abilities) available. Our application showed that we need different composite factors to predict grades in mathematics and English. For English grades, the composite factor was essentially determined by the facet verbal analogies, whereas a linear combination of all three facets predicted mathematics grades. From the perspective of criterion-related validity, it might not always be best to rely on *g* as an underlying variable (“property of a test”) but to use the best combination of test scores for a specific purpose, which might be viewed as the best exploitation of the available information. 

The first-order factor model can be reformulated to a model with a reflective general factor on which all observed indicators load. In such a bifactor(*S*-1) model, the first-order factor of a facet taken as reference facet defines the common factor. The indicators of the non-reference specific abilities are regressed on the reference factor. The specific part of a non-reference facet that is not determined by the common reference factor is represented by a specific factor. The specific factors can be correlated. If one puts certain restrictions on the parameters in the bifactor(*S*-1) model, as done in the application, the model is data equivalent to the first-order factor model (for a deeper discussion see [48]). The main difference to the first-order factor model is that the regression weight of the reference facet factor (the common factor) does not depend on the other facets (in a regression model predicting criterion variables). The regression weight equals the regression coefficient in a simple regression analysis, because the reference factor is uncorrelated with all other factors. However, the regression coefficients of the remaining facets represent that part of a facet that does not depend on the reference facet. Depending on the reference facets chosen the regression weights of the specific factors might differ. Because the specific factors can be correlated a regression coefficient of a specific factor indicates the contribution of the specific factor beyond the other specific factors (and the reference facet). 

The bifactor(*S*-1) model is particularly useful if a meaningful reference facet exists. For example, if an intelligence researcher aims to contrast different facets of intelligence against one reference facet (e.g., fluid intelligence) that she or he considers as basic, the bifactor(*S*-1) model would be the appropriate model. For example, Baumert, Brunner, Lüdtke, and Trautwein [51] analyzed the cognitive abilities assessed in the international PISA study using a nested factor model which equals a bifactor(*S*-1) model. They took the figure and word analogy tests as indicators of a common reference intelligence factor (analogies) with which verbal and mathematical abilities (represented by a specific factor respectively) were contrasted. The common intelligence factor had a clear meaning (analogies) that is a priori defined by the researcher. Therefore, researchers are aware of what they are measuring. This is in contrast to applications of *g* models in which specific factors have zero variance as a result of the analysis. For example, Johnson, Bouchard, Krueger, McGue, and Gottesman [52] could show that the *g* factors derived from three test batteries were very strongly correlated. They defined a *g* factor as a second order factor for each test battery. In the model linking the three test batteries, each *g* factor has a very strong loading (1.00, 0.99, 0.95) with a verbal ability facet. Given these high factor loadings, there is no room for a specific factor for verbal abilities and *g* essentially equals verbal abilities. Therefore, the three very strongly related *g* factors were three verbal ability factors. Johnson, te Nijenhuis, and Bouchard [53] could confirm that the *g* factors of three other test batteries were also strongly correlated. In their analysis, the three *g* factors were most strongly linked to first-order factors assessing mechanical and geometrical abilities. Consequently, the meaning of the *g* factors might differ between the two studies. The meaning of *g* has always been referred to from looking at complex loading structures and often it reduces to one stronger reference facet. An advantage of a priori defining a reference facet has the advantage that the meaning of the common factor is clear and can be easily communicated to the scientific community. The empirical application presented in this paper showed that verbal analogies might be such an outstanding facet for predicting school grades. If one selects this facet as the reference facet, the specific factors of the other facets do not contribute to predicting English grades, but they contribute to mathematics grades. 

## 5. Conclusions and Recommendations

Given the identification and estimation problems, the utility of the bifactor model for predicting criterion variables by general and specific factors is questionable. Further research is needed to scrutinize under which conditions a bifactor model with additional correlating criterion variables can be appropriately applied. At the very least, when the bifactor model is applied to analyze correlations with general and specific factors, it is necessary to report all correlations and regressions weights as well as their standard errors in order to decide whether or not the bifactor model was appropriately applied in a specific research context. In applications in which the correlations of some specific factors with criterion variables are fixed to 0 and are not reported, it remains unclear whether one would not have also found a well-fitting model with substantive correlations for all specific factors and non-significant correlations for the general factor. In the current paper, we recommend applying two alternative models, first-order factor models and bifactor(*S*-1) models. The choice between first-order factor models and bifactor(*S*-1) models depends on the availability of a facet that can be taken as reference. If there is a meaningful reference facet or a facet that is of specific scientific interest, the bifactor(*S*-1) model would be the model of choice. If one does not want to make a distinction between the different specific facets, the first-order factor model can be applied. 

## Figures and Tables

**Table 1:** Correlations between Observed Variables.

	*NS*_1_	*NS*_2_	*AN*_1_	*AN*_2_	*UN*_1_	*UN*_2_	Math	Eng
*NS*_1_	4.456							
*NS*_2_	0.787	4.487						
*AN*_1_	0.348	0.297	4.496					
*AN*_2_	0.376	0.347	0.687	4.045				
*UN*_1_	0.383	0.378	0.295	0.366	5.168			
*UN*_2_	0.282	0.319	0.224	0.239	0.688	5.539		
Math	0.349	0.350	0.289	0.378	0.302	0.275		
Eng	0.225	0.205	0.263	0.241	0.135	0.097	0.469	
Means	4.438	3.817	4.196	4.018	4.900	4.411		
Proportions of the grades							1: 0.123 2: 0.311 3: 0.297 4: 0.174 5: 0.096	1: 0.059 2: 0.393 3: 0.338 4: 0.174 5: 0.037

Note. Variances of the continuous variables are given in the diagonal. *NS_i_* = number series, *AN_i_* = verbal analogies, *UN_i_* = unfolding, *i* = test half, Math = mathematics grade, Eng = English grade.

**Table 2:** Bifactor Model and Grades.

	*G*-Factor Loadings	*S*-Factor Loadings	Residual Variances	Rel	Covariances						
						*G*	*NS-S*	*AN-S*	*UN-S*	Math	Eng
*NS*_1_	1 **0.651**	1 **0.615**	0.882 (0.176) **0.198**	0.802	*G*	1.887 (0.481)	0	0	0	*0.286*	*0.150*
*NS*_2_	0.971 (0.098) **0.630**	1 **0.613**	1.022 (0.199) **0.228**	0.772	*NS-S*	0	1.687 (0.331)	0	0	*0.272*	*0.194*
*AN*_1_	0.759 (0.161) **0.492**	1 **0.620**	1.681 (0.255) **0.374**	0.626	*AN-S*	0	0	1.726 (0.316)	0	*0.283*	*0.270*
*AN*_2_	0.838 (0.162) **0.573**	1 **0.653**	0.993 (0.217) **0.245**	0.755	*UN-S*	0	0	0	2.207 (0.441)	*0.212*	*0.058*
*UN*_1_	1.000 (0.199) **0.604**	1 **0.653**	1.074 (0.215) **0.208**	0.792	Math	*0.393* (0.456)	*0.353* (0.445)	*0.371* (0.353)	*0.315* (0.428)		
*UN*_2_	0.781 (0.198) **0.456**	1 **0.631**	2.181 (0.334) **0.394**	0.606	Eng	*0.206* (0.470)	*0.252* (0.475)	*0.355* (0.384)	*0.086* (0.460)	0.469 (0.055)	

Notes. Parameter estimates, standard errors of unstandardized parameter estimates (in parentheses), standardized parameter estimates (bold type). Covariances (right side of the table) are presented below the diagonal, variances in the diagonal, and correlations above the diagonal. Rel = reliability estimates, *NS_i_* = number series, *AN_i_* = verbal analogies, *UN_i_* = unfolding, *i* = test half, Math = mathematics grade, Eng = English grade. All parameter estimates are significantly different from 0 (*p* < 0.05) with the exceptions of parameters that are set in italics.

**Table 3:** Multivariate Regression Analyses with the Mathematics and English Grades as Dependent Variables and the *g* Factor and the Three Specific Factors as Independent Variables.

	Mathematics (*R*^2^ = 0.284)		English (*R*^2^ = 0.113)	
	*b*	*b_s_*	*B*	*b_s_*
*G*	0.205 (0.234)	0.282	0.115 (0.246)	0.158
*NS-S*	0.213 (0.264)	0.276	0.143 (0.283)	0.186
*AN-S*	0.218 (0.207)	0.286	0.200 (0.223)	0.264
*UN-S*	0.145 (0.198)	0.216	0.035 (0.208)	0.051

Notes. Regression parameter estimates (*b*), standard errors of unstandardized regression parameter estimates (in parentheses), standardized regression estimates (*b_s_*), and coefficient of determination (*R*^2^). *G =* general factor, *NS-S* = number series specific factor, *AN-S* = verbal analogies specific factor, *UN-S =* unfolding specific factor, Math = Mathematics grade, Eng = English grade. None of the estimated parameters are significantly different from 0 (all *p* > 0.05).

**Table 4:** Estimates of the Model with Correlated First-order Factors and Grades.

	Factor Loadings	Residual Variances	Rel	Covariances					
					*NS*	*AN*	*UN*	Math	Eng
*NS*_1_	1 **0.889**	0.938 (0.200) **0.211**	0.789	*NS*	3.519 (0.425)	0.464	0.461	0.394	0.242
*NS*_2_	1 **0.886**	0.967 (0.197) **0.215**	0.785	*AN*	1.490 (0.274)	2.927 (0.394)	0.408	0.400	0.304
*AN*_1_	1 **0.807**	1.569 (0.290) **0.349**	0.651	*UN*	1.661 (0.302)	1.338 (0.277)	3.680 (0.493)	0.349	0.142
*AN*_2_	1 **0.851**	1.118 (0.257) **0.276**	0.724	Math	0.740 (0.127)	0.685 (0.126)	0.669 (0.134)		0.469
*UN*_1_	1 **0.844**	1.487 (0.365) **0.288**	0.712	Eng	0.455 (0.136)	0.520 (0.128)	0.272 (0.133)	0.469	
*UN*_2_	1 **0.815**	1.859 (0.390) **0.336**	0.664						

Notes. Parameter estimates, standard errors of unstandardized parameter estimates (in parentheses), and standardized parameter estimates (bold type). Covariances (right side of the table) are presented below the diagonal, variances in the diagonal, and correlations above the diagonal. Rel = reliability estimates, *NS_i_* = number series, *AN_i_* = verbal analogies, *UN_i_* = unfolding, *i* = test half, Math = mathematics grade, Eng = English grade. All parameter estimates are significantly different from 0 (*p* < 0.05).

**Table 5:** Multivariate Regression Analyses with Mathematics and English Grades as Dependent Variables and the Three Intelligence Factors as Independent Variables.

	Mathematics (*R*^2^ = 0.233)		English (*R*^2^ = 0.106)	
	*b*	*b_s_*	*b*	*b_s_*
*NS*	0.113 ** (0.039)	0.213	0.073 (0.046)	0.137
*AN*	0.140 ** (0.046)	0.239	0.146 ** (0.050)	0.250
*UN*	0.080 * (0.037)	0.153	−0.012 (0.041)	−0.023

Notes. Regression parameter estimates (*b*), standard errors of unstandardized regression parameter estimates (in parentheses), standardized regression estimates (*b_s_*), and coefficient of determination (*R*^2^). *NS* = number series, *AN* = verbal analogies, *UN* = unfolding, Math = Mathematics grade, Eng = English grade. ** *p* < 0.01, * *p* < 0.05.

**Table 6:** Bifactor(*S*-1) Model with Correlated First-order Factors and Grades.

	*G*-Factor Loadings	*S*-Factor Loadings	Residual Variances	Rel	Covariances					
						*NS-S*	*AN*	*UN-S*	Math	Eng
*NS*_1_	0.509 (0.083) **0.412**	1 **0.787**	0.938 (0.200) **0.211**	0.789	*NS-S*	2.760 (0.333)	0	0.337	0.235	*0.114*
*NS*_2_	0.509 (0.083) **0.411**	1 **0.784**	0.968 (0.197) **0.216**	0.784	*AN*	0	2.928 (0.394)	0	0.400	0.304
*AN*_1_	1 **0.807**		1.568 (0.290) **0.349**	0.651	*UN-S*	0.980 (0.244)	0	3.069 (0.442)	0.203	*0.020*
*AN*_2_	1 **0.851**		1.117 (0.257) **0.276**	0.724	Math	0.391 (0.110)	0.685 (0.126)	0.356 (0.124)		
*UN*_1_	0.457 (0.084) **0.344**	1 **0.771**	1.487 (0.365) **0.288**	0.712	Eng	*0.190* (0.121)	0.520 (0.128)	*0.035* (0.123)	0.469 (0.055)	
*UN*_2_	0.781 (0.084) **0.332**	1 **0.744**	1.858 (0.390) **0.336**	0.664						

Notes. Parameter estimates, standard errors of unstandardized parameter estimates (in parentheses), and standardized parameter estimates (bold type). Covariances (right side of the table) are presented below the diagonal, variances in the diagonal, and correlations above the diagonal. Rel = reliability estimates, *NS_i_* = number series, *AN_i_* = verbal analogies, *UN_i_* = unfolding, *i* = test half, *AN =* verbal analogies reference facet factor, *NS-S* = number series specific factor, *UN-S =* unfolding specific factor, Math = Mathematics grade, Eng = English grade. All parameter estimates are significantly different from 0 (*p* < 0.05) with the exceptions of parameters that are set in italics.

**Table 7:** Multivariate Regression analyses with the Mathematics and English Grades as Dependent Variables and the Three Factors of the Bifactor(*S*-1) Model as Independent Variables (Reference Facet = Verbal Analogies).

	Mathematics (*R*^2^ = 0.233)		English (*R*^2^ = 0.106)	
	*b*	*b_s_*	*b*	*b_s_*
*AN*	0.234 ** (0.038)	0.400	0.178 ** (0.040)	0.304
*NS-S*	0.113 ** (0.046)	0.188	0.073 (0.046)	0.122
*UN-S*	0.080 * (0.037)	0.140	−0.012 (0.041)	−0.021

Note. Regression parameter estimates (*b*), standard errors of unstandardized regression parameter estimates (in parentheses), standardized regression estimates (*b_s_*), and coefficient of determination (*R*^2^). *AN* = verbal analogies reference facet factor, *NS-S* = number series specific factor, *UN-S* = unfolding specific factor, Math = Mathematics grade, Eng = English grade. ** *p* < 0.01, * *p* < 0.05.

**Table 8:** Multivariate Regression analyses with the Mathematics and English Grades as Dependent Variables and the Three Factors of the Bifactor(*S*-1) Model as Independent Variables (Reference Facet = Number Series).

	Mathematics (*R*^2^ = 0.233)		English (*R*^2^ = 0.106)	
	*b*	*b_s_*	*b*	*b_s_*
*NS*	0.210 ** (0.031)	0.394	0.129 ** (0.037)	0.242
*AN-S*	0.140 ** (0.046)	0.212	0.146 ** (0.050)	0.221
*UN-S*	0.080 * (0.037)	0.136	−0.012 (0.041)	−0.021

Note. Regression parameter estimates (*b*), standard errors of unstandardized regression parameter estimates (in parentheses), standardized regression estimates (*b_s_*), and coefficient of determination (*R*^2^). *NS* = number series reference facet factor, *AS-S* = verbal analogies specific factor, *UN-S* = unfolding specific factor, Math = Mathematics grade, Eng = English grade. ** *p* < 0.01, * *p* < 0.05.

## Footnotes

1 For reasons of parsimony, we present standard errors and significance tests only for unstandardized solutions (across all analyses included in this paper). The corresponding information for the standardized solutions leads to the same conclusions.

2 From a historical point of view this early paper is also interesting for the debate on the role of general and specific factors. It showed that achievements in school subjects that do not belong to the science or language spectrum such as shops and crafts as well as drawing were more strongly correlated with the specific spatial ability factor (*r* = 0.461 and *r* = 0.692) than with the general factor (*r* = 0.219 and *r* = 0.412), whereas the *g* factor was more strongly correlated with all other school domains (between *r* = 0.374 and *r* = 0.586) than the specific factor (between *r* = −0.057 and *r* = 0.257).

## Appendix A

In the text, it is shown that a bifactor model with a correlating criterion variable is not identified if the indicators do not differ in their loading parameters. In this appendix, it will be shown that a bifactor model with a correlating criterion variable is identified if the loadings on the general factor differ. We only refer to the covariance structure. In all models of confirmatory factor analysis, either one loading parameter per factor or the variance of the factor has to be fixed to a positive value to get an identified model. We chose the Mplus default setting with fixing one loading parameter per factor to 1. Because there are only two indicators per specific factor and the specific factors are not correlated with the remaining specific factors, we fixed all factor loadings of the specific factors to 1. Whereas the nonidentification of bifactor models with equal loadings refers to all bifactor models independently of the number of indicators and specific facets, the identification of models with freely estimated loadings on the general and specific factors depends on the number of indicators and specific factors. The proof of identification of the bifactor model with correlating criterion variables in general goes beyond the scope of the present research and will not be provided. We only consider the models applied in the empirical application.

In the following, a general factor is denoted with *G*, the facet-specific factors are denoted with *S_k_*, the observed variables with *Y_ik_*, and measurement error variables with *E_ik_*. The first index *i* refers to the indicator, the second indicator *k* to the facet. Hence, *Y*_11_ is the first indicator of the first facet considered. A criterion variable is denoted with *C*. We consider only one criterion variable. We only consider models in which the criterion variables are correlated with the factors. Because the regression coefficients in a multiple regression model are functions of the covariances, the identification issues also apply to the multiple regression model. Moreover, we will only consider the identification of the covariances between the criterion variables and the general as well as specific factors because the identification of the bifactor model itself has been shown elsewhere (e.g., [54]). In the models applied, it is assumed that the criterion variables are categorical variables with underlying continuous variables. The variables *C* are the underlying continuous variables. If the criterion variable is a continuous variable, *C* denotes the continuous variable itself. In the model with free loadings on the general factor, the observed variables can be decomposed in the following way:

$$\;Y_{ik}=\lambda_{ik}G+S_{k}+E_{ik}\;$$

with *λ*_11_ = 1. The covariance of an observed variable *Y_ik_* with the criterion can be decomposed in the following way:

$$\;Cov(Y_{ik},C)=Cov\left(\lambda_{ik}G+S_{k}+E_{ik},\;C\right)=\lambda_{ik}Cov\left(G,C\right)+Cov\left(S_{k},C\right)\;$$

with

$$\;Cov(Y_{11},C)=Cov\left(G+S_{1}+E_{11},\;C\right)=Cov\left(G,C\right)+Cov\left(S_{1},C\right)\;$$

For the difference between the two covariances $Cov(Y_{11},C)$ and $Cov(Y_{21},C)$ the following decomposition holds:

$$\;Cov(Y_{11},C)-Cov(Y_{21},C)=Cov\left(G,C\right)+Cov\left(S_{1},C\right)-\lambda_{21}Cov\left(G,C\right)-Cov\left(S_{1},C\right)\;=Cov\left(G,C\right)-\lambda_{21}Cov\left(G,C\right)=\left(1-\lambda_{21}\right)Cov\left(G,C\right)\;$$

Consequently, the covariance between the general factor and the criterion variable is identified by

$$\;Cov\left(G,C\right)=[Cov(Y_{11},C)-Cov(Y_{21},C)]/\left(1-\lambda_{21}\right)\;$$

with

$$\;\lambda_{21}=Cov(Y_{21},Y_{12})/Cov(Y_{11},Y_{12})\;$$

The covariances between the three specific factors and the criterion variable are identified by the following equations:

$$\begin{array}{c} \;Cov\left(S_{1},C\right)=Cov(Y_{21},C)-\;\lambda_{21}Cov\left(G,C\right)=Cov(Y_{21},C)-\frac{Cov(Y_{21},Y_{12})\left[Cov(Y_{11},C\right)-Cov(Y_{21},C)]}{Cov(Y_{11},Y_{12})\left(1-Cov(Y_{21},Y_{12}\right)/Cov(Y_{11},Y_{12}))}\\ \;Cov\left(S_{2},C\right)=Cov(Y_{12},C)-\;\lambda_{12}Cov\left(G,C\right)=Cov(Y_{21},C)-\frac{Cov(Y_{12},Y_{13})\left[Cov(Y_{11},C\right)-Cov(Y_{21},C)]}{Cov(Y_{11},Y_{13})\left(1-Cov(Y_{21},Y_{12}\right)/Cov(Y_{11},Y_{12}))}\\ \;Cov\left(S_{3},C\right)=Cov(Y_{13},C)-\;\lambda_{13}Cov\left(G,C\right)=Cov(Y_{13},C)-\frac{Cov(Y_{13},Y_{12})\left[Cov(Y_{11},C\right)-Cov(Y_{21},C)]}{Cov(Y_{11},Y_{12})\left(1-Cov(Y_{21},Y_{12}\right)/Cov(Y_{11},Y_{12}))}\; \end{array}$$
